# An Exploration of Victim Blaming and Bystander Intervention in the Context of Image-Based Sexual Abuse: A Scoping Review

**DOI:** 10.3390/bs16050757

**Published:** 2026-05-12

**Authors:** Loren E. Parton, Michaela M. Rogers

**Affiliations:** School of Sociological Studies, Politics and International Relations, University of Sheffield, Sheffield S10 2AH, UK; m.rogers@sheffield.ac.uk

**Keywords:** cyber abuse, non-consensual image sharing, non-consensual porn, sexual image-based abuse, scoping review, technology-facilitated violence and abuse

## Abstract

This scoping review synthesises the current literature to explore the related concepts of victim blaming and bystander intervention in the context of image-based sexual abuse. Image-based sexual abuse refers to the creation, taking and distribution of non-consensual intimate images, including the threat to share or distribute. The databases Web of Science, ASSIA, ProQuest Dissertation & Theses and Scopus were searched in August 2024, with an updated search being conducted in December 2025. A supplementary search was conducted in Google Scholar, along with a hand search of four key journals within the topic area. The search focused on five geographical locations that share a common cultural background (UK, USA, Canada, New Zealand, and Australia). A total of 31 studies and reviews were included. The main findings were that: (a) there is limited research in relation to bystander intervention in the context of image-based sexual abuse; (b) there are no studies that examine the relationship between victim blaming and bystander intervention; (c) there appears to be a gendered dimension in relation to the phenomena (victim blaming and bystander intervention), which is reflected in the literature around image-based sexual abuse; (d) accountability and victim blaming are increased when a victim–survivor has created the images/videos themselves; (e) research within this area neglects the experiences of diverse communities, specifically sexual and gender minority people; and (f) there appears to be a disregard to capture the experiences of men who are victim–survivors, irrespective of sexual identity.

## 1. Introduction

Image-based sexual abuse (IBSA) is defined as the creation, taking and distribution of non-consensual intimate images, including the threat to share or distribute (Henry & Beard, 2024). IBSA is an umbrella term that encompasses a wide range of behaviours, from the creation and non-consensual distribution of intimate images to upskirting to sextortion (Henry & Powell, 2018; Patchin & Hinduja, 2020). It also includes the unsolicited sharing of sexually explicit images, known informally as ‘dick pics’, as well as the use of artificial intelligence (AI) to create fake, sexualised images, known as ‘deepfakes’ (Flynn et al., 2022). The common denominator across these behaviours relates to the use of media, images or videos in order to harm others; hence, the term ‘image-based sexual abuse’ is adopted in this review as an overarching term that captures complex and multifaceted phenomena. McGlynn et al. (2017) argue that IBSA should be conceptualised as a continuum of behaviours. This is useful and evocative of a dynamic umbrella category that can accommodate the ever-changing and growing range of harmful behaviours facilitated by the use of technology. At the same time, this allows for different modes of IBSA to be theorised separately or in relation to one another, as well as a consideration of the overall harms of IBSA collectively. As such, IBSA is a form of sexualised psychological violence characterised by a loss of control, public exposure and a persistent threat.

Although the literature suggests that IBSA perpetration and victimisation are becoming increasingly common amongst adults and young people (Powell et al., 2019), there still remain challenges around varied applications of terminology and differences in methodologies. The term ‘revenge pornography’, which is frequently adopted in this field, is problematic in that the use of the words ‘porn’ or ‘pornography’ has connotations of consent and material produced for sexual entertainment. Furthermore, the term suggests a sole motivation of revenge, implying the victim–survivor is somehow to blame for the perpetrator’s need to enact revenge. Research suggests that the motivation for this act is significantly more complex (Maddocks, 2018) and includes the desire for control over another person, and to cause psychological harm and humiliation, rather than for mere revenge (Parton & Rogers, 2025). In terms of methodologies, research focusing on sensitive topics can be a challenge in terms of recruitment and samples; for example, students are convenient because a large sample is readily available in one geographical location. This is the case for the current scholarship on IBSA, in that a sizable number of published studies have adopted student samples, which can affect the conclusions and generalisability of the results found.

In terms of the impact of IBSA, current scholarship highlights a range of consequences for victim–survivors, including physical and mental health and social and financial impacts. Much of this scholarship is nascent, as, for example, prior to 2016, there was no published literature on the psychological and mental health impacts of IBSA for victim–survivors. Addressing this gap, Bates (2017) focused on the mental health impacts experienced and coping mechanisms of victim–survivors of IBSA. The results showed that women described emotional and mental health effects, such as post-traumatic stress disorder, anxiety, depression, and self-esteem issues. She also identified social and relational impacts, such as trust issues and loss of control, in relation to their victimisation. In terms of negative coping mechanisms, these included excessive alcohol consumption, self-medication and denial. Short et al. (2017) found that half of their participants (N = 64) used self-harm to alleviate negative thoughts and feelings related to victimisation.

Demonstrating the medium- and long-term impacts of IBSA, Rackley et al.’s (2021) research highlighted that the majority of victim–survivors had experienced abuse that had occurred within the last 5 years, while for some participants, IBSA had occurred over 10 years ago, and they were still experiencing negative consequences. Specifically, Rackley et al. (2021, p. 298) described the ways in which several participants disconnected from the online and offline world as ‘social rupture’. A. Huber (2023) contextualised the impact of IBSA victimisation as not an isolated event but as part of the behaviours that women exhibit in their everyday lives. She highlighted a range of medium- and long-term outcomes in relation to social wellbeing, health, employment and financial stability. A. Huber (2023) suggested that fear of further victimisation becomes ingrained in victim–survivors’ everyday behaviour, thus highlighting the complexity of longer-term impacts of IBSA.

Paradoxically, the reporting of IBSA has been identified as negatively exacerbating the experience and causing further harm to victim–survivors (Rackley et al., 2021). The differing legislation depending on the country in which the offence occurs feeds into the harms and impact experienced by victim–survivors due to inconsistencies in relation to prosecution. Moreover, legislation is continuously modified, in line with emerging technologies and changes in their use and misuse. In the UK, for example, recent amendments have been made to the Online Safety Act 2023 (UK Parliament, 2023), with new offences being introduced into the Sexual Offences Act 2003 (UK Parliament, 2003). This includes a base offence of sharing an intimate image without consent or reasonable belief in consent (Women and Equalities Committee, 2025). A further update in September 2024 to the Online Safety Act 2023 (UK Parliament, 2023) has designated such offences as ‘priority offences’, meaning that platform providers have a greater responsibility to remove and prevent harmful content.

Similarly, countries such as Australia have been suggested to be leading the way in terms of the country’s responsiveness to IBSA legislation and the protection of victim–survivors. In contrast, not all states in the USA have legislation to address IBSA, and criminalisation varies from state to state (Cyber Civil Rights Initiative, 2021). Finally, both New Zealand and Canada introduced legislation in 2014/2015; however, this is not specific to IBSA and focuses on online abuse more generally. The current legislation across each of the countries appears to be both confusing and restrictive in that it does not appropriately consider the range of behaviours associated with IBSA; for example, some legislation fails to consider the creation of intimate images. Furthermore, if the sharing of intimate images occurs within the context of an intimate relationship and relates to coercive or controlling behaviour, this then falls under different legislation (Serious Crime Act 2015 (UK Parliament, 2015)).

This lack of consistency in legal frameworks feeds into the narrative of victim–survivors being blamed or responsibilised (McGlynn et al., 2017) for experiencing IBSA in that if these type of offences go unpunished due to difficulty in prosecution or do not reach the prosecution stage due to trivialisation of the behaviours by professionals, the idea of victim–survivors being at fault may be a convenient conclusion to make. These issues serve to impede disclosures and help-seeking, resulting in further harm to victim–survivors and potentially long-term impacts, which inevitably hinder processes of recovery.

## 2. Conceptual Framework and Current Scholarship

**Figure 1:**
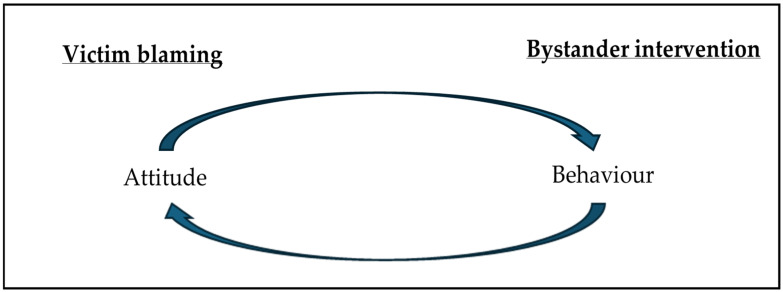
A guiding framework to propose a multidirectional relationship between victim blaming and bystander intervention.

The conceptual framework for this review examines two related research concepts: victim blaming (attitude) and bystander intervention (behaviour). In combination, these concepts help to examine the influence of relevant attitudes and behaviours that may exist in terms of attributing accountability in cases of IBSA, as well as reporting by and help-seeking of victim–survivors of IBSA. Figure 1 demonstrates a tentative guiding model that underpins this review and provides a clear presentation of how these two concepts dynamically relate to one another. The diagram proposes that there is a multidirectional relationship between the concept of victim blaming (attitude) and shows how it influences bystander intervention (behaviour), and vice versa. We combine these concepts because in scholarship, it is reported that a barrier to intervention is the presence of victim-blaming attitudes and the attribution of accountability to those who experience sexual violence and abuse (Stoner & Cramer, 2019). We also note that victim blaming can be adopted to justify the decision not to intervene.

### 2.1. Victim Blaming

The term ‘victim blaming’ was coined by Ryan (1971) and refers to the process in which the individual who has experienced harm is held accountable for the crime committed against them. Beddows (2022) argues that this term is narrow and restrictive in that the process that is experienced by victim–survivors involves more than just being blamed. Alternatively, Beddows advocates for the adoption of Barry’s (1979) concept of ‘victimism’, which proposes that when women are given victim status, they are reduced to this label and their experiences, stripped of their agency, rather than being treated as a whole person. This concept considers the wider structural issues of patriarchy and how the system produces and maintains victim status.

The theme of victim blaming and its related concept, accountability, are commonly depicted in social media and public contexts, as well as within academic research (Clay, 2019). In the UK, numerous police-led campaigns, such as ‘Party animals’ and ‘Safe night out’, appear to detract from the perpetrators of sexual crimes and attribute blame and responsibility to the victim–survivors (Dymock, 2017). Similarly, in Australia, a short video called ‘Megan’s story’ has been used to highlight the risks of sexting behaviour but also criticised for focusing on the outcome for the victim–survivor and levelling blame at Megan (Albury & Crawford, 2012). In the USA, a study about sexual violence prevention amongst college students found that many of the suggestions placed responsibility on the potential victim rather than targeting preventative strategies at potential perpetrators, thus also feeding into the victim-blaming narrative (Berdera & Nordmeyer, 2015). The responsibilisation of victim–survivors in these campaigns is enacted through the emphasis on the need for their behaviour change to ensure their own safety, rather than on the behaviour change of perpetrators in terms of desistance. The majority of such campaigns have been problematised by anti-victim-blaming organisations and quickly removed. This is significant as victim blaming can impede help-seeking and reporting in cases of sexual violence, as victim–survivors are less likely to formally report their experiences due to the fears of victim-blaming reactions from professionals and services (Anderson & Overby, 2021). Indeed, research has highlighted that these attitudes are held by those employed in supportive roles, such as police and support services (Naezer & van Oosterhout, 2021; O’Neal, 2019).

Research by Hatcher (2016) found that rape myth acceptance and victim infidelity were predictive of ‘revenge porn’ victim blaming. Furthermore, contextual variables such as the nature of the relationship can influence victim blaming; for example, higher levels of victim blaming were attributed when the perpetrator was an ex-partner, in contrast to the least blame being attributed when the perpetrator was a stranger (Krahé et al., 2007). Like many other forms of sexual abuse, the issue is tied to a cultural system that values women’s chastity and supports men’s promiscuity (Chisala-Tempelhoff & Kirya, 2016). Therefore, rather than addressing IBSA as a form of sexual violence, women are advised to simply stop taking sexually explicit images of themselves or allowing others to do so (Bloom, 2014). As such, women are blamed for behaving counter to societal norms rather than perpetrators being held to account for their harmful behaviour.

### 2.2. Bystander Intervention

The second concept in this conceptual framework is that of a bystander, alternatively known as a ‘digital bystander’, who, in the context of IBSA, can be defined as an individual who views an intimate image by means of non-consensual sharing (Difranzo et al., 2018; Harder, 2021). Research within this area initially started by exploring offline spaces, such as during emergency incidents, where there was a low rate of bystander involvement, with inquiries aimed at identifying the potential rationale behind bystanders choosing to intervene or not in situations. Scholarship now centres on online spaces, and consistently low rates of bystander intervention have been found (Bhandari et al., 2021).

The bystander effect refers to the impact of the presence of others on an individual’s decision to intervene in a situation (Dovidio et al., 2017). This can relate to concepts of diffusion of responsibility, an individual feeling that someone else may help and a reduced sense of responsibility. Additionally, an individual may look to others for social cues; thus, if others do not respond, this may encourage the individual not to intervene either. Latané and Darley’s (1970) model, which refers to what we know today as the bystander effect, includes five stages that are suggested to either encourage intervention or deter individuals from becoming involved in a situation. These include: an individual actually recognising an incident; interpreting this as an emergency; taking responsibility for engaging with the emergency; deciding how to act; and, finally, choosing to act. If barriers occur within these five stages, this could impact the bystander’s choice to intervene (Burn, 2009).

Predominantly, research in this area has focused upon inhibitors and facilitators of bystander intervention (Rizzo et al., 2022) and sexual violence (Mainwaring et al., 2022). Bystander intervention has received limited exploration in terms of the context of IBSA, but it has been gaining more traction. At present, there have been no reviews exploring the interactions between the two concepts: victim blaming and bystander intervention in relation to IBSA. This area of research remains limited; thus, the current scoping review aims to explore the current knowledge gap in the literature as a foundation to establish whether there is a relationship between these concepts. The findings of this review have the potential to inform the understanding of how attitudes may contribute to behaviour, as well as contribute to interventions aimed at responding to both the victimisation and perpetration of online harms. In this context, bystander intervention might include actions such as the formal reporting of inappropriate shared material online to law enforcement or online platforms. It might include confronting or naming abusive acts in online forums directly to the person posting images.

## 3. Methods

The aim of a scoping review is to identify research gaps in the existing literature and synthesise research findings in an attempt to answer specific research questions (Arksey & O’Malley, 2005). Furthermore, it serves to clarify key concepts and definitions, as well as to consider the key factors associated with concepts within the research questions. Research by Munn et al. (2018) suggests that these are some of the main purposes for conducting a scoping review. As these align with the rationale for the current study, this methodology was deemed appropriate. The review was conducted in accordance with the Preferred Reporting Items for Systematic Reviews and Meta-analyses extension for Scoping Reviews (PRISMA-ScR) guidelines to ensure transparency and reliable reporting of the literature (Tricco et al., 2018). A five-stage approach was adopted as per Arksey and O’Malley’s (2005) recommended methodological framework for scoping reviews: (1) identifying research questions, (2) identifying relevant studies, (3) selecting studies, (4) charting data, and finally, (5) collating, summarising and reporting the results. The decision was made not to exclude studies on the basis of quality (Arksey & O’Malley, 2005) because the aims of this review were to explore the current landscape around the above-mentioned concepts in the context of IBSA, and excluding relevant articles and data may impact the validity of the results. This has been deemed an appropriate approach amongst other scoping reviews, for example, Pham et al. (2014). The review protocol was registered at the Open Science Framework on 16 January 2026 [https://osf.io/3fbz7].

### 3.1. Research Questions

This scoping review presents an overview of the current body of literature across five countries (UK, USA, Canada, New Zealand and Australia) in relation to the concepts of victim blaming and bystander intervention in the context of IBSA. The scoping review questions are:

1. How do empirical studies conceptualise and operationalise victim blaming in the context of IBSA?
2. Which forms of bystander intervention are examined in IBSA, and what facilitators/inhibitors are identified?
3. What harms are associated with victim blaming and bystander intervention in the context of IBSA, and how are these harms operationalised?
4. Does current scholarship explore victim blaming and bystander intervention as related concepts in the context of IBSA?

### 3.2. Search Strategy

The following databases were searched in August 2024: Web of Science, ASSIA and Scopus. Supplementary searches were conducted using Google Scholar and ProQuest Dissertation & Theses Global to ensure an exhaustive search of unpublished dissertations and the grey literature. Finally, four key journals within this area of scholarship, namely, ‘*Violence Against Women*’, ‘*Sexual Abuse*’, ‘*Journal of Interpersonal Violence*’ and ‘*Journal of Sexual Aggression*’, were hand searched to identify any further relevant literature. An updated search was conducted in December 2025 to ensure that all relevant studies were captured and the review was as up to date as possible. The search terms used to explore the literature in relation to the defined research questions are presented in Table 1.

The selection criteria include type of study, sample characteristics, date range and publication language (see Table 2 for inclusion and exclusion criteria). The review was limited to countries that have shared socio-cultural contexts, thus being able to provide comparative discussions of the results found. Furthermore, a recent review conducted by Afrouz and Vassos (2024) on a similar research theme did not exclude research by country but found that the final sample of included studies was more prevalent in the UK, USA, Canada, New Zealand and Australia. We include only English-language publications to match the language ability of the research team and because this project was unfunded, meaning no support for additional resources.

**Figure A1:**
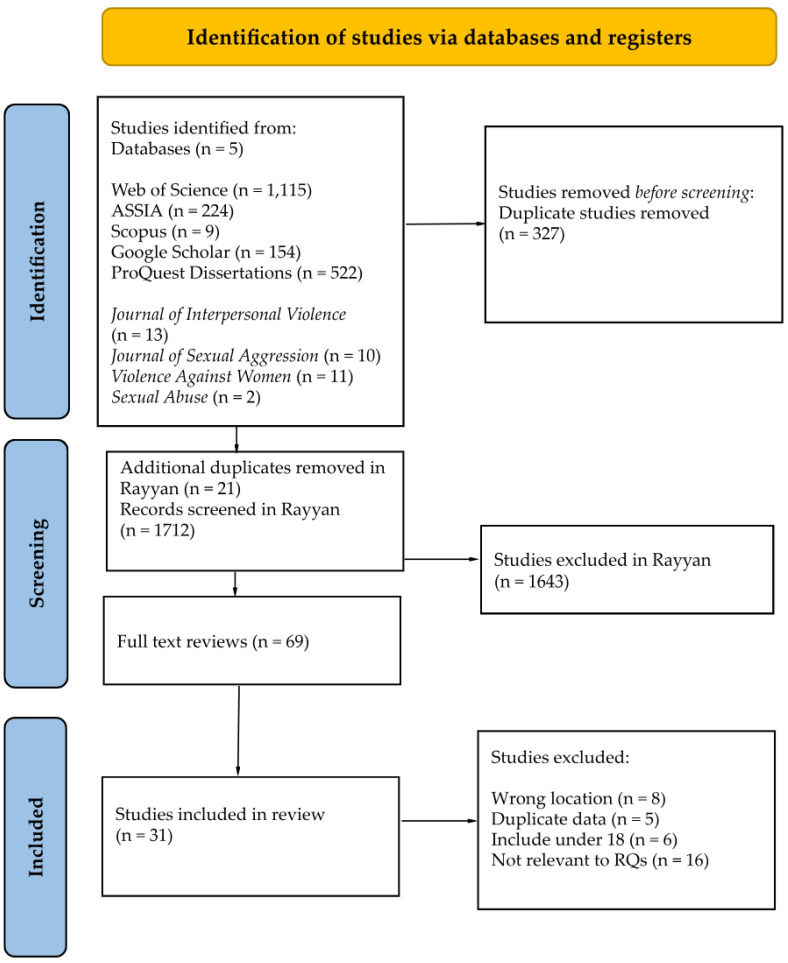
PRISMA-ScR flowchart of search strategy.

The initial search generated 2060 articles, with 1731 articles remaining after initial duplicates were removed. The software Rayyan was utilised by both researchers, in which blind reviewing was conducted with the initial number of articles (*n* = 1712). Any further duplicates were removed (*n* = 21), and discrepancies were then resolved through discussion prior to the next stage of the review. In total, 1643 articles were excluded based on titles and abstracts, and 37 were excluded following full-text reading. Overall, 31 articles were retained in the final analysis. Following the initial screening process, both researchers screened the same 10 articles to ensure reliability across the decision-making process and then equally screened half of the remaining articles to determine if they should be included or excluded from the final review. Again, discussions resolved conflicts, and all decisions made were appropriately recorded. A PRISMA-ScR flow chart (Figure A1 in Appendix A) was created to present the stages of data selection for the scoping review.

### 3.3. Data Extraction

A data extraction template (see Table A1 in the Appendix B) was developed in order to synthesise the findings of each of the included articles. The extracted data included author(s), year of publication, origin/country of origin (where the source was published or conducted), population and sample size within the source of evidence, methodology and key findings that relate to the scoping review question/s. Some articles include duplicate data, in that both published articles and theses have been included in the search strategy. Therefore, this is noted on the data extraction table for transparency and has not been included in the final number of articles.

## 4. Results

The final stage of this scoping review involved summarising and reporting the results, and a data extraction table was used as a guide to explore the findings of the articles with an aim to address the proposed research questions. This section of this paper begins with a description of the included studies, including the location of the studies as well as the methodologies chosen by the authors. Following this, each subsequent subsection focuses on addressing each of the proposed research questions. Under each main heading, key themes are discussed, which relate to characteristics such as gender, age and sexual orientation. When discussing the included studies under each theme, the terminology utilised within each of the studies is adopted to demonstrate the wide range of vocabulary used in this area. This includes the use of the term ‘revenge porn(ography)’, which does not reflect the views held by the authors, but instead is included as this is the original terminology used in the cited papers within this review. Due to the volume of articles included, it was not possible to include the full data extraction table within the main body of this review. For this information, please refer to Appendix B.

### 4.1. Description of Included Studies

The 31 included studies primarily used quantitative research methods (N = 18), with nine adopting qualitative methods and two a mixed-methods approach. The majority of the studies were empirical (N = 27), with only four being reviews or a mix of both empirical and systematic reviews. A high proportion of the included articles did focus on using experimental methods, such as the use of vignettes (N = 13). The studies were mainly from the UK (N = 11) and the USA (N = 8), with the other studies being from Australia (N = 5), Canada (N = 3), and New Zealand (N = 1), and one study was based on data from a combination of the UK, Australia and New Zealand. Predominantly, the samples used within each of the studies were either general population samples (N = 17), students (N = 5) or those who had experienced IBSA (N = 4). However, there were six studies that included data from stakeholders, such as those working in victim services, police, activists, policy and government organisations and criminal justice staff. Although the studies each relate to one or more of the explored phenomena (victim blaming and bystander intervention), 16 different terms are used to describe IBSA or a specific behaviour characterised by IBSA. The most common terminology used is revenge porn (N = 9), followed by IBSA (N = 7). Other terms such as sextortion (N = 2), cyberflashing (N = 1), non-consensual porn (N = 2) and technology-facilitated violence and abuse (N = 1) are used.

### 4.2. Victim Blaming and Bystander Intervention

This section firstly highlights a number of factors that relate to characteristics and/or demographics of victim–survivors, which have been suggested to make an individual more susceptible to holding victim-blaming attitudes or to experiencing victim blaming themselves. Furthermore, it highlights the presence of victim blaming within support services and by the police. These factors are as follows: gender, sexual orientation, consent and accountability, relationship factors and attitudes of support services. Secondly, it considers the inhibitors and facilitators that influence bystander intervention. The final section explores the harms associated with victim blaming and bystander intervention. The final research question asks whether victim blaming and bystander intervention have been explored as related concepts; however, as there were no studies that investigated this, this has been discussed further in the Discussion and Conclusion Sections.

### 4.3. Gender

The included studies suggest that gender has an influence on the attribution of blame to a victim–survivor, with men attributing more blame to victim–survivors in comparison to women (Attrill-Smith et al., 2021; Bothamley & Tully, 2017; Rhyner, 2017; Woolford, 2019). Morris (2017) found this gendered distinction in relation to victim blaming, as well as evidence that men displayed more empathy for the perpetrator, especially when the victim–survivor was a woman. In relation to the specific offence of ‘upskirting’, Fido et al. (2023) found that participants attributed lower levels of victim blame to vignettes including victim–survivors who were women as opposed to those vignettes in which victim–survivors were men. Flynn et al. (2023) found that men were more likely to blame victim–survivors of IBSA if the individuals had taken and/or sent the intimate image of themselves; this was in comparison to women and sexually diverse communities. In their systematic review, Amudhan et al. (2024) similarly found that men attribute more blame towards victim–survivors who are women, particularly when the intimate image was self-taken.

In contrast to the suggestion that victim blaming is gendered (Flynn et al., 2023; Morris, 2017), Chatzisymeonidis and Pina (2024) found that police officers who are women exhibited higher levels of victim blaming in contrast to officers who are men. However, on closer examination, this was not found to be statistically significant. The authors suggest that this difference may reflect that not all types of crime are subject to gender-based stereotypes, in that men hold higher victim-blaming attitudes than women. Additionally, the context and environment in which the offence occurred, in this case, an ‘online space’ may impact victim-blaming attitudes. Ragen (2023) hypothesised that men would exhibit higher victim-blaming attitudes than women; however, the opposite was found. This result could be explained by the additional factor of religious fundamentalism, which was assessed during the study, and the belief that a moral code has been broken; hence, the higher levels of victim blaming. In relation to men who are victim–survivors of IBSA, Gavin and Scott (2019) found that these experiences are often minimised and trivialised. Similarly, Uhl (2017) found that participants viewed the offence as more severe when a woman had taken a photograph of themselves in contrast to the victimisation experience of a man who had done the same.

Flynn et al. (2022a) examined the role of bystanders in preventing IBSA in Australia using a survey and focus groups. The focus group included the use of hypothetical scenarios that depicted witnessing IBSA in different contexts. The results showed that when the gender of the victim–survivor and/or perpetrator was changed, for example, male perpetrator, female victim–survivor or female perpetrator, male victim–survivor, gender did influence an individual’s willingness to intervene. The results from female participants suggested that they were less likely to intervene due to safety concerns if the perpetrator was a man. This is similar to responses from male participants, who suggested that if the perpetrator were a woman, they would also be less likely to intervene due to established gender roles and feeling it would not be appropriate to intervene.

A second article published from Flynn et al.’s (2022a) study examined specifically how gender affected bystander experiences and responses using the survey results. They found that gender was significant in impacting an individual’s intervention when witnessing abuse, with women more likely to take no action than men. However, there was no discussion on whether this was influenced by the gender of the victim–survivor and/or perpetrator (Flynn et al., 2022b). In contrast, Krieger (2020) examined factors that would influence a bystander’s intervention in relation to receiving a non-consensual sexual image, finding that women were more likely to intervene in the behaviour and assigned less blame to the victim–survivor. This study, however, did not explore whether the gender of the victim–survivor and/or perpetrator influenced a participant’s decision to intervene in the witnessed behaviour.

### 4.4. Sexual Orientation

C. R. Serpe (2020) explored victim blaming against sexually diverse populations (bisexual, lesbian and heterosexual adults). This study asked heterosexual, cisgender men and women to complete an online survey after engaging with vignettes that depicted sexually diverse women as victim–survivors of revenge porn. The results suggested that there was no significant relationship in regard to the sexuality of the victim–survivor. Similarly, Bradbury (2023) found that sexual orientation did not impact the amount of blame placed on depicted victim–survivors of IBSA. Flynn et al. (2023) collected responses from heterosexual men and women, as well as sexually diverse individuals who they categorised as lesbian, gay or bisexual (LGB+), in relation to victim-blaming attitudes. The results suggested that heterosexual men were more likely to blame victim–survivors if they had taken and/or sent the intimate image of themselves, in contrast to heterosexual women. Similarly, LGB+ individuals were less likely to blame victim–survivors; however, they did score higher than both heterosexual men and women on acceptable exceptions, meaning that they find it acceptable to share unsolicited intimate images or images of strangers.

### 4.5. Consent and Accountability

In their study of university students, Gavin and Scott (2019) found that victim-blaming attitudes were associated with the notion of implied consent, in that participants felt that by taking intimate images or ‘selfies’, an individual should have known the risks of them being shared. Moreover, participants indicated that victim–survivors should have taken steps to mitigate this risk, thus perceiving IBSA victim–survivors as accountable for the consequences of their actions. This study was focused specifically on the context of sending intimate images within a romantic relationship; thus, the focus was on consenting to this during the period of the relationship. This reflects the findings of studies by Mclocklin et al. (2024), Attrill-Smith et al. (2021) and Rhyner (2017), who found that when an individual created content themselves, such as intimate images or videos, more blame was assigned to them, highlighting the attribution of accountability upon victim–survivors rather than highlighting the behaviours of perpetrators. Similarly, a systematic review conducted by Amudhan et al. (2024) found that victim–survivors were often blamed and labelled immoral, particularly if they had taken an intimate image themselves, rather than considering the contextual factors of the behaviour, such as an intimate image being shared without consent. Gosse (2022) proposed the term ‘responsibilisation’, suggesting that victim–survivors are expected to take on the responsibility of avoiding and preventing their abuse, highlighting the societal attitude that pushes blame onto victim–survivors rather than making the perpetrators of this abuse culpable. This is further supported by Zauner (2021), who analysed ‘sexting’ education campaigns and found that a key theme within these campaigns was victim–survivors being held accountable for their own victimisation and that they are responsible for assessing the risk of a situation before engaging with it. Furthermore, Krieger’s (2020) study found that there was a significant positive correlation between victim blaming and victim responsibility and that men attributed more blame and responsibility to victim–survivors than female participants. In this particular study, the victim–survivor was a woman who had taken an intimate ‘selfie’. Finally, Cowley (2024) found that women who were sex workers were suggested to be more culpable for being victim–survivors of TFSV than non-sex workers, thus being accountable due to their profession.

### 4.6. Relationship Factors: Infidelity/Perceived Promiscuity

Victim–survivors who are perceived as being more promiscuous—for example, depicted in images wearing minimal clothing—experience higher levels of blame (Mckinlay & Lavis, 2020). Similarly, Bradbury (2023) found that perceptions, whether a victim–survivor was perceived as promiscuous or not, were influenced by the sexual orientation of the victim–survivor; for example, gay and lesbian victim–survivors were perceived as more promiscuous than their heterosexual counterparts. Additionally, victim–survivors who were perceived as more promiscuous were perceived as being more to blame for their victimisation. This suggests the existence of bias within this particular sample, in that non heterosexual men experience higher levels of victim blaming because participants perceive them as more promiscuous than heterosexual men. Morris (2017) found that when a victim–survivor was presented as having a history of infidelity, participants displayed a more negative reaction to the victim–survivor and were more likely to empathise with the perpetrator.

### 4.7. Relationship Factors: Length of Relationship

Using scenarios that depicted revenge porn, Starr and Lavis (2018) considered whether the length of a relationship and level of trust predicted the perceptions of blame and betrayal. Their results showed that individuals were blamed less when the length of the relationship was longer (1 year) in comparison to a short relationship (1 month). This is explained by the perceived level of betrayal and trust: if a relationship is more long-term, trust is more established, thus a victim–survivor of revenge porn in this context would be blamed less than if they were in a new relationship, where trust would not have been yet solidified, hence being perceived as more blameworthy. This contrasts with findings by Bothamley and Tully (2017), who found no significant relationship between the length of a relationship and victim-blaming attribution. However, it is important to highlight that in their study, they did not specifically define the length of a relationship and used the terms ‘a short while’ and ‘a long time’, so participants had to form their own perception of the length of the relationship. The authors suggested that if the length of time was specified, the perception of whether it was a long or short relationship would differ; thus, it may impact the results, as it was not controlled for.

### 4.8. Attitudes of Support Services

Henry et al. (2018) examined the perspectives of stakeholders, which included law enforcement, academics, policy experts and sexual violence advocates, to explore the current responses to IBSA in Australia and the barriers and challenges that are occurring around this offence. They identified a number of obstacles, with victim-blaming attitudes being particularly relevant to this article. A range of stakeholders identified that a lack of understanding around gendered violence, as well as victim-blaming attitudes by the police, contributed to the low reporting rates.

Importantly, an individual’s diversity background was highlighted as a barrier to reporting, specifically LGBTIQ+ communities and adults with disabilities. Henry et al. (2018) found that there is a lack of trust in these communities, specifically in relation to previous negative experiences, as well as minimisation and the fear of not being believed or taken seriously; hence, the low reporting rates of IBSA. Similarly, A. R. Huber (2020) and Gauthier (2023) found that victim–survivors of IBSA did experience victim blaming from the police, which led to barriers in reporting the offence to the police due to the fear of being blamed for the creation of images. Additionally, the results suggested that for the majority of victim–survivors, the response from the police was inappropriate and ineffective, with many victim–survivors feeling they were not being taken seriously, or their needs were not being met. Likewise, a study examining police perceptions of technology-facilitated intimate-partner violence found that the police were more likely to attribute blame to victim–survivors who delayed reporting the offence to them, again suggesting the idea of victim–survivors being almost responsible and/or accountable for the behaviour that has occurred against them (Chatzisymeonidis & Pina, 2024).

### 4.9. Factors That Impact Bystander Intervention

This review found that research into the role of bystanders in the context of IBSA was limited in terms of the number of existing studies on this phenomenon. One study by Flynn et al. (2022a) did identify that bystander intervention is a common occurrence within this context, with it being suggested that two-thirds of 245 participants in their study had witnessed IBSA.

Studies by Mainwaring et al. (2024, 2025) drew on data collected as part of Mainwaring’s (2023) thesis, which explored the individual, situational and contextual facilitators and barriers to an individual intervening when witnessing IBSA. These included victim empathy, confidence, being friends with the victim–survivor and fears for safety and audience inhibition. These have all been suggested within previous research exploring bystanders and sexual violence (Labhardt et al., 2017; Mainwaring et al., 2022). A factor that appears to be specific to the IBSA context relates to the influence of self-taken images, in that individuals are more likely to blame the victim–survivors because they are responsible for the creation of the image, as well as being less supportive of prosecution and criminalisation of the behaviour. Furthermore, Mainwaring (2023) found that bystanders were less likely to intervene when the image was self-taken, suggesting that perceptions of consent or ownership of an image are influential factors in bystander intervention, particularly within the context of online abuse. A scoping review conducted by Kromann and Flynn (2025) found that intervention was reduced when bystanders perceived the victim–survivor as having high sexual agency, such as a willingness to take intimate pictures. Each of these studies suggests that rather than intervening and supporting victim–survivors in these scenarios, bystanders were more likely to judge victim–survivors for engaging in sexting or taking intimate images rather than holding the perpetrator accountable for their wrongdoing.

### 4.10. Harms

In terms of judgements held in regard to IBSA, research suggests that it is not as harmful as physical sexual abuse (du Mello Gibbard & Fido, 2023). This suggests the perception of IBSA as not as serious or that it does not have as many short- and long-term effects as other forms of sexual violence. Research by Flynn et al. (2023) found that heterosexual men were more likely to minimise the harms of IBSA; furthermore, those individuals who actually engaged in taking explicit images of themselves also held attitudes of minimisation around the impact of IBSA. Jeacock et al. (2024) explored the behaviour of cyberflashing to understand the experiences of victim–survivors. Their results suggested that cyberflashing was not perceived as serious by victim–survivors, but there was the recognition that it was non-consensual and did cause a negative impact on them. Furthermore, one of the key themes from the interviews was around victim blaming and that many victim–survivors felt they were to blame for perpetrators sending them explicit images. Fido et al. (2023) looked at perceptions of upskirting and suggested that the experiences and harms of men may be viewed less seriously than those of their counterparts who are women. Additionally, male victim–survivors may be ‘written out of the victimisation narrative’ due to the use of terminology (that is, ‘upskirting’) and labelling of this offence (Fido et al., 2023, p. 17).

On the other hand, some studies did acknowledge that IBSA was as harmful as other forms of abuse. Gohil and Fido (2021) found that participants recognised IBSA as a crime and highlighted the long-term effects/impact of being a victim–survivor of this offence; these included issues around trust, suicide, withdrawal and anxiety. Additionally, a review by Amudhan et al. (2024) found that when victim–survivors were blamed or responsibilised, this resulted in negative psychological impacts, limited help-seeking behaviour from support services and exacerbated stigma around these types of offences. Furthermore, Kugler and Pace (2021) found that participants perceived that the creation and dissemination of ‘deepfake’ videos was as harmful as the dissemination of ‘traditional’ non-consensual intimate images.

## 5. Discussion

In this scoping review, 31 studies were included that related to victim blaming and bystander intervention in the context of IBSA and the associated effects of these concepts. Perhaps one of the most significant findings is that the synthesis of evidence suggests a relationship between victim blaming and bystander intervention in the context of IBSA. However, it is acknowledged that there were no studies found that directly explored this relationship; thus, this finding should be examined with caution. Therefore, the authors do not suggest a causal relationship but instead two related concepts that are underpinned by shared theoretical links. Several studies found that accountability was implicated along with the attitude that victim–survivors of IBSA are somewhat responsible for their actions. Although introduced by sociologist Nicolas Rose in 2000, more recently, the concept of responsibilisation has gained traction in sexual violence scholarship, with Hadjimatheou (2022, p. 321) defining it as “the attribution of causal and even moral responsibility for abuse to victim-survivors”. Responsibilising victim–survivors of IBSA problematically overlooks “the structural and institutional constraints that limit the possibilities for safety and freedom” (Coy & Kelly, 2019, p. 4) whilst simultaneously absolving others (that is, bystanders) of responsibility for women’s safety. It reinforces gender norms and stereotypes that problematically position women as victims, rather than survivors, or victim–survivors, and reduces their ‘space for action’ (Kelly et al., 2014) to seek support and redress in cases of IBSA.

Further, the studies in this review that focused on victim–survivors who had taken images of themselves, or ‘selfies’, found that these individuals were seen as more to blame (Attrill-Smith et al., 2021; Gavin & Scott, 2019; Gosse, 2022; Rhyner, 2017; Zauner, 2021). This is supported by a recent empirical study by Adair and Senn (2025) that was not included in this review because it did not meet the inclusion criteria. However, interestingly, the study found the situational context of an image (being taken or shared by the victim–survivor themselves) influenced the attribution of blame, in that victim–survivors were perceived as more to blame in contrast to those whose images had been shared or taken non-consensually. A finding from a systematic review conducted by Amudhan et al. (2024) supports the narrative that if women are seen as having high sexual agency, they experience more blame for the outcome of their actions.

Furthermore, Kromann and Flynn (2025) found that victim–survivors being perceived as having higher sexual agency directly influenced the actions of bystanders, in that they were less likely to intervene when witnessing IBSA. Despite the subsequent non-consensual sharing of these images, because of the implications that victim–survivors had consented and been complicit in creating an intimate picture, the subsequent sharing of the picture was viewed as a direct consequence of the victim–survivor’s own actions. The attribution of blame in this context ultimately influenced bystander decision-making in terms of whether or not to take action. Indeed, research by Mainwaring (2023) found that the notion of a woman taking a ‘selfie’ and being active in the creation of her own sexualised image was a specific inhibitor in bystander action within the context of IBSA. In this context, Gosse (2022) highlights how responsibilisation serves to shift accountability and blame to the victim–survivor as they are deemed to have ‘caused’ this behaviour, rather than rightly attributing the wrongdoing to the perpetrator. This finding is consolidated in other research, such as Krieger’s (2020) study, which uncovered a direct positive correlation between victim blaming and victim responsibility. The presence of responsibilisation in relation to IBSA highlights an important finding in terms of the attribution of accountability and blame. It also highlights issues around consent in relation to sexual images, in that there is a clear need for public consensus that consent to share an image must always be gained from the person who is the focus of a sexualised image when that image was created within the context of a trusting intimate relationship, whatever the status of that relationship.

There are, of course, other psychological and cognitive reasons why bystanders decide not to intervene in cases of IBSA, including the lack of recognition that bystander action is warranted or ignorance that what they have observed is morally inappropriate or even unlawful, instead interpreting what they see as funny or harmless (Parton & Rogers, 2025). In addition, bystanders can absolve themselves of responsibility to act by holding the view that someone else will. Alternatively, the decision not to intervene might be due to a fear of retaliation from the parties involved.

Second, the stark lack of representation of the experiences of diverse communities in IBSA research, specifically LGBTQI+ people, has implications in terms of conceptual understandings of IBSA and also in relation to policy and practice responses. It was well reported that sexual and gender minority people are often invisibilised or marginalised in the wider field of domestic, family and sexual violence research, theorising, policy and practice (McSpadden, 2024; M. Rogers, 2020). In the current review, only four studies included or distinguished LGBTQI+ experiences. Flynn et al. (2022a) compared the experiences of heterosexual and LGB+ participants; however, this was still not representative, as sexually diverse participants were examined as one group due to insufficient data available to conduct a reliable analysis. They did consider gender differences in the experiences and responses of bystanders in the context of IBSA; however, they focused specifically on the responses from men and women, rather than people of any gender identity (Flynn et al., 2022b). The research team attempted to explore the gaps in knowledge around LGBTQI+ communities and included a trans woman in their bystander scenarios. The responses from the focus groups related to participants’ desire to intervene more in the situation due to ongoing challenges and discrimination faced by marginalised communities, rather than due to the behaviour occurring.

It is essential that these experiences are captured, as research suggests that adults from the LGBTQI+ communities experience disproportionately higher levels of digital harassment and abuse, as well as sexual, sexuality-based and gender-based harassment, when compared to heterosexual cisgender adults (Powell et al., 2020). Further research into LGBTQI+ people’s experiences of IBSA is critical to address this gap and to advance understanding that can quash concepts such as responsibilisation in the context of minority communities. By adopting this term in the context of marginalised communities, it shifts responsibility onto individuals to manage their own harm and frames disproportionate levels of abuse as a result of individual behaviour rather than structural discrimination (Donovan & Hester, 2010). Furthermore, when these communities do seek help, the heteronormative design of research, policy and services means that their experiences are misunderstood and not appropriately responded to, thus creating a ‘double-bind’ situation (Donovan & Barnes, 2019). There was a distinct lack of scholarship that reflects other social categories and identities, such as age, disability or religion.

Third, the review found a gendered dimension within the relationship between victim blaming and bystander intervention. The results suggest that men appear to hold more victim-blaming attitudes in the context of IBSA (Attrill-Smith et al., 2021; Bothamley & Tully, 2017; Rhyner, 2017). However, studies that examined bystander intervention suggested mixed results in terms of gender, in that some studies found that women were more likely to take no action when witnessing IBSA (Flynn et al., 2022b). However, in contrast to this, Krieger (2020) found that women were more likely to intervene than men. However, this study is particularly limited by not exploring how the gender of the victim–survivor and/or perpetrator may influence or change bystander decision making.

Due to the limited number of studies that discuss bystander intervention within this scoping review, no concrete conclusions can be drawn from these findings, which highlights the clear gap in this research area. Furthermore, in a number of the studies, the experiences by men appear to be trivialised or minimised, in that participants reported that the impacts of IBSA are not as bad for men in contrast to the experiences of women (Fido et al., 2023; Gavin & Scott, 2019; Uhl, 2017). This indicates a lack of research in this field at present that captures the experiences of men who are victim–survivors, whatever their sexual identity. This is reflected in some of the study designs that used vignettes or scenarios as a means to collect data, in that they included heteronormative and binary depictions of victim–survivors as women and perpetrators as men (Krieger, 2020; Starr & Lavis, 2018; Zauner, 2021). This heteronormative bias means there could be difficulties in generalising the results of studies to scenarios such as victim–survivors who are men and perpetrators who are women, or in the case of same-sex couples.

The findings highlighted the limited research on bystander intervention that has been discussed above in the context of IBSA. A further consideration regarding the lack of research may relate to issues around IBSA legislation and its inconsistency across the five countries this review has explored, in that it does not always encompass the full range of behaviours associated with IBSA. This, in turn, could influence victim–survivors reporting of offences, as well as following through with prosecution, limiting the knowledge and understanding of this topic area.

Fourth, the review found that the concept of help-seeking was examined in relation to the influences on victim–survivors seeking support following IBSA from professional services and criminal justice agencies. One of the barriers identified by both Henry et al. (2018) and A. R. Huber (2020) was the presence of victim-blaming attitudes held by professionals, including the police, and how these attitudes reduced reporting rates. This finding reflects the wider body of sexual violence research, rather than IBSA scholarship in particular (Anderson & Overby, 2021; Naezer & van Oosterhout, 2021; O’Neal, 2019). This suggests challenges not only in relation to individual attitudes but within the context of systemic and structural challenges, in that attitudes are embedded within organisations and institutions. A review by M. M. Rogers et al. (2022) highlighted that practitioners do not feel confident or knowledgeable in responding to technology-facilitated abuse, which is highly problematic in the face of the ever-increasing and diverse uses of technology. The lack of professional knowledge and confidence also suggests a reduction in the ‘conducive context’ (Kelly, 2007) critically needed for victim–survivors to feel safe enough to disclose IBSA, obtain appropriate support and seek legal redress. When using an intersectional lens, it is recognised that there are additional barriers to reporting and accessing support when victim–survivors identify with a minority social category, whether this relates to gender, sexual orientation, ethnicity or other. This has further implications for creating the conducive context necessary for minoritised people to be able to disclose safety and seek support in cases of IBSA. In terms of legal remedies and frameworks, there is a lack of robust research that has examined IBSA and legislation.

Finally, in terms of the harms and/or outcomes of IBSA, the review found that these were impactful and long-lasting. These included social, financial, relational, psychological and mental health outcomes (Gohil & Fido, 2021; Kugler & Pace, 2021). This reflects the impacts of IBSA found in the work by Henry et al. (2019). However, some studies found that IBSA is not considered as serious or as harmful as other forms of sexual violence, strengthening the argument that responsibilisation serves to hold women to account in their experiences of IBSA, as they are blamed for creating sexualised images in the first place, whilst simultaneously reducing the accountability attributed to perpetrators (Jeacock et al., 2024; Kugler & Pace, 2021). The temporal dimension of the harms experienced by victim–survivors is important, as many only find out that their images have been shared after the creation of the image or after the relationship has ended and, therefore, consent has not been given. This again highlights the continued debate around IBSA sitting on a continuum of sexual violence (Kelly, 1988), in that the harms mirror other forms of sexual violence, and thus, it should be treated similarly in terms of legislation and available support services. However, the prevalent discourse around IBSA is that it is perceived as less serious and less harmful because it is perpetrated in an online space; thus, drawing on Kelly’s (1988) hierarchy of harms, it may be considered as less legitimate and impactful. This, in turn, can influence the availability of service provisions and specialist services, as well as the legislative response. This has an even deeper impact on marginalised groups when considering Kelly’s hierarchy, in that these communities are placed lower as they do not fit the typical ‘heteronormative’ depiction of a victim–survivor.

### 5.1. Methodological Considerations

This review draws sharply into focus the problem of multiple terms adopted in both similar and different ways to describe phenomena that come under the umbrella of IBSA. An important point to consider is that ten studies used the terminology ‘revenge porn/pornography’, and within current IBSA scholarship, the adoption and application of the term ‘revenge porn’ is particularly challenging and an unsuitable term to describe the non-consensual sharing of sexual images (Parton & Rogers, 2025). In addition, it is possible that the use of this now-familiar terminology may have influenced the views and perceptions of the study participants, in that it can encourage the adoption of victim-blaming attitudes and minimisation. Exploring the influence of language in future research would help to determine the impact on perceptions and judgments dependent on the language adopted.

Almost half of the included studies (N = 14) used vignettes or scenarios as their chosen methodology. Due to this, consideration needs to be given to whether vignettes are representative of the normative perceptions and attitudes of an individual. Hughes and Huby (2002) suggest that vignettes have been extensively used within social research, the advantage being that they can overcome ethical challenges when accessing particularly hard-to-reach participant groups as well as undertaking research on sensitive topics. These are also useful in researching new and emerging behaviours. However, there are limitations in that vignettes cannot completely capture real-life experiences, which raises the question of how generalisable results and conclusions are outside of the vignette scenario.

### 5.2. Limitations

A limitation of this review relates to the methodology, as although a robust search strategy was employed (Tricco et al., 2018), there is a risk that some articles that met the inclusion criteria were not included in this review, as we limited the number of databases searched. However, as the purpose of this scoping review is to assess the current landscape of the literature on this topic, this will not impact the ability of the authors to draw conclusions.

A second limitation relates to the inclusion criteria, in that only adults (18+) were included in this review. Some articles were excluded if the sample included people under 18 years who could not be distinguished from the 18+ sample. There were two articles excluded on this basis. Although research suggests that IBSA is a common phenomenon in young people (see Ringrose et al., 2021), this can present alternative challenges and differing research contexts due to the consideration of these types of behaviours/offences classified as child sexual offences. The author’s research focus for this review was to consider IBSA in relation to the adult population (people aged 18+).

## 6. Conclusions

This scoping review aimed to explore two phenomena, victim blaming and bystander intervention, and how these may relate to each other in the context of IBSA. To the authors’ knowledge, this is the first scoping review to consider these phenomena as related to one another, rather than in isolation. Although the 31 included studies explored these concepts, no studies examined them together or how they may or may not influence each other. As with previous studies examining IBSA (Parton & Rogers, 2025), the identity category of gender was considered in much of the research, but it was heteronormative in its focus, and there is limited research that considers the experiences of diverse groups, specifically LGBTQI+ communities, in relation to victim blaming and bystander intervention. Many of the studies included considered responsibilisation and how accountability was shifted onto victim–survivors for their suggested role in their victimisation by taking intimate images of themselves. Furthermore, research would benefit from exploring this concept further, along with other highlighted concepts of victimism and victim blaming, to understand how effective they are in the context of IBSA victim–survivors. This will be particularly important in the context of IBSA due to victim–survivors engaging in ‘selfie-taking’ behaviour. Finally, a high proportion of the included studies focused on the use of vignettes (N = 14). Future research should use in-depth qualitative inquiry to capture the experiences of diverse communities to further our understanding of IBSA.

## Figures and Tables

**Table 1:** Search terms.

Issue	Outcome
Image-based sexual abuse	Harm *
Revenge porn *	Impact *
Technology facilitated sexual abuse	Consequence *
Technology facilitated violence and abuse	Outcome *
Non-consensual sharing of intimate images	Reporting
Bystander effect *	Flagging
Victim blam *	
Bystander intervention	
Upstander behaviour	

Please note, asterisks have been used within the search terms to act as wildcard placeholders to broaden the search perimeters by including searches for phrase variations.

**Table 2:** Inclusion and exclusion search criteria.

Criteria	Inclusion	Exclusion
Types of sources	Primary research (qualitative and quantitative), case studies, reviews, grey literature, theses and reports	Non-empirical studies, theoretical articles, legal analyses, book chapters and book reviews
Date	Publications between 2000 and 2025	Publications prior to 2000
Language	Publications in English UK, USA, Canada, Australia and New Zealand	Non-English publications Other countries
Demographic	Adults 18+ Victim–survivors Perpetrators Professionals/practitioners	Sample that includes adolescents/children under 18 years old
Concepts	Include discussion on either one or both concepts of victim blaming and bystander intervention in the context of IBSA	Studies that do not include any of the concepts of victim blaming and bystander intervention in the context of IBSA

## Data Availability

No new data were created or analysed in this study. Data sharing is not applicable to this article.

## Appendix B

**Table A1:** Data extraction summary of included studies.

Author/s (Year)	Country of Origin/Author Location	Sample (N, Characteristics)	Methodology	Finding/Propositions
Amudhan et al. (2024)	International	N = 10 studies.	Systematic review—Qualitative	This review explored the literature on victim blaming and non-consensual pornography. They found that a number of psychosocial factors and culture influenced blaming of victim–survivors. The review found that gender influenced victim blaming, particularly in relation to gender stereotyping and there being greater responsibilisation on women who experience this type of abuse.
Attrill-Smith et al. (2021)	UK	N = 342 adults (76 male, 266 female). General population age 18–68 years.	Online video evaluations and survey—Quantitative	This article explores gender differences in victim-blaming attitudes towards revenge porn. Participants were shown videoed accounts of real experiences of revenge porn. These videos included stories of revenge porn, based on celebrity videos and reports, which were re-told by actors. The video accounts included both male and female victim–survivors, and self- and stealth-taken sexual videos and images. The results show that when participants were asked directly, they were more likely to blame the perpetrator than the victim–survivor. However, when blame was assigned to victim–survivors, more blame was assigned to those who generated the material themselves, and male participants were more likely to blame victim–survivors than their female counterparts.
Bothamley and Tully (2017)	UK	N = 168 adults (52 males, 116 females). General population age 19–71 years.	Online survey using a vignette approach—Quantitative	This article investigates public perceptions of revenge porn and victim blaming, specifically in relation to the length of the perpetrator–victim relationship, the necessity of police intervention and harm. The findings show that the length of the perpetrator–victim relationship did not affect participants’ attribution of blame to victim–survivors. Participants agreed that incidents of revenge porn were likely to cause psychological harm to the victim–survivor, and that police intervention was somewhat necessary. Male participants were more likely to blame the victim–survivor, and female participants were more likely to view police intervention as necessary.
Bradbury (2023)	Australia	N = 178 adults (69 males, 106 females). General population age 18–73 years.	Online survey using a vignette approach—Quantitative	This thesis examines whether sexual orientation, perceived promiscuity and morality affect how people assign blame in cases of revenge porn. The results show that the victim–survivor’s sexuality did not impact the levels of blame assigned to them by participants. However, perceptions of promiscuity did differ based on the victim–survivor’s sexuality.
Chatzisymeonidis and Pina (2024)	UK	N = 108 police officers in the UK (51 male, 57 female).	Online survey using a vignette approach—Quantitative	This article explores how police officers recognise and respond to cyberstalking. Additionally, it investigates the factors influencing police officers’ attribution of blame to victim–survivors. The results show that police officers who had received specialised intimate-partner violence training were more likely to recognise the case presented in the vignette as cyberstalking, whilst those who had not were more unsure. When victim–survivors delayed reporting, police officers were more likely to attribute blame to them.
Cowley (2024)	New Zealand/International sample	N = 221 men age 18–75 years.	Online survey—Quantitative	This thesis explores victim blaming in the context of technology facilitated sexual violence (TFSV). It includes two studies: The first examines how Rape and Sexual Aggression Myth beliefs influence victim blaming. The results show that women engaged in sex work were more likely to be blamed for sexual harassment and TFSV. Study two investigates the effect of masculinity threat on victim culpability attributions in the context of TFSV. The findings show that masculinity threats had limited influence on victim-blaming attitudes, but hostile sexism was associated with higher attributions of victim culpability following such threats.
du Mello Gibbard and Fido (2023)	UK	N = 76 adults (21 male, 55 female). General population. Mean age given: 29.75 years.	Online survey—Quantitative	This article compares judgements of image-based sexual abuse (IBSA) to those of physical sexual abuse. The results indicate that participants had more lenient judgements of vignettes that depicted IBSA. Additionally, endorsement of beliefs about revenge pornography led to more lenient judgements of IBSA, but not of physical sexual abuse.
Fido et al. (2023)	UK	N = 490 adults (250 male, 240 female). General population. Mean age given: 39.42 years.	Online survey using a vignette approach—Quantitative	This article explores social judgements and proclivities to commit upskirting. Specifically, it investigates whether judgements differ depending on the sex of the victim–survivor and their perceived attractiveness. The results show that more lenient judgements were given when the victim–survivor was attractive and male. The article also examines the extent to which variation in voyeuristic interest, belief in a just world, and dark personality traits impact judgements and proclivity to engage in upskirting. Findings suggest that proclivity to engage in upskirting was predicted by voyeuristic behaviours and higher psychopathic personality.
* Flynn et al. (2022a)	Australia	Survey: N = 245 adults (73 male, 161 female, 11 non-binary or other). General population. Age 18–71 years. Focus groups: N = 219 adults (from the original sample of 245 adults).	Online survey and face-to-face focus groups—Mixed methods	This article investigates the role of bystanders in preventing image-based abuse (IBA). The results show that among participants, witnessing IBA was common (two-thirds had witnessed IBA). Yet, only 45.6% of participants who provided further information about their most recent experience witnessing IBA took action against it. The study also revealed significant gender differences in interventions—women were more likely to not take action.
Flynn et al. (2023)	UK, Australia, Aotearoa/New Zealand	N = 43 stakeholders.	Interviews with stakeholders—Qualitative	This article examines attitudes of blame and minimisation of harm in relation to IBSA. Interviews with stakeholders revealed that victim-blaming attitudes and harm minimisation are linked to sexual double standards in society, as well as problematic attitudes surrounding sexual violence in general.
Gauthier (2023)	Canada	N = 175 sources that document perceptions of image-based sexual harassment and abuse.	Systematic review—Qualitative	This thesis investigates perceptions of IBSHA via a systematic review. The findings reveal that the way in which IBSHA is structured can lead to negative attitudes towards victim–survivors. These results demonstrate the need for policy-orientated and intervention practices to help improve attitudes and responses to victim–survivors of IBSHA.
Gavin and Scott (2019)	UK	N = 222 UK university students (111 male, 111 female).	Questionnaire—mixed methods, but only the qualitative data is presented in the article	This article explores victim blaming in relation to revenge porn. The results suggest that the majority of participants attributed some level of blame to the victim–survivor in the scenarios of revenge porn depicted. Furthermore, the authors suggest that victim-blaming attitudes are related to the implied consent of sharing intimate images with a partner. They also suggest that the experiences of male victim–survivors are often trivialised.
Gosse (2022)	Canada	N = 15 women who had experienced online abuse. Age 21–44 years.	Semi-structured interviews—Qualitative	This article demonstrates how victim–survivors of online abuse (including the sharing of intimate images or videos) are responsibilised—they are expected to take on responsibility for avoiding and preventing their abuse. This process of responsibilisation involves victim blaming and self-blame, normalisation, minimisation and attempts to control the risk of abuse and its related harms.
Henry et al. (2018)	Australia	N = 52 Australian stakeholders (12 male, 39 female and 1 transgender participant).	Interviews with Australian legal and policy experts, domestic and sexual violence advocates, industry representatives, police and academics—Qualitative	This article investigates stakeholders’ responses to IBSA in Australia. The authors suggest that IBSA has begun to be treated more seriously by the police, but there are still barriers to reporting. These barriers include: inconsistent laws, a lack of resources, evidentiary limitations, jurisdictional boundaries, victim blaming and harm minimisation attitudes. The authors therefore suggest that improvements could be made to the criminal justice space, including the provision of more tailored police training as well as the provision of more culturally sensitive responses to victim–survivors.
A. R. Huber (2020)	UK	N = 17 victim–survivors of IBSA (age 19–46 years, all female). N = 6 activists. N = 5 criminal justice staff.	In-depth interviews with victims, activists and criminal justice staff—Qualitative	This thesis takes a radical feminist and victimological approach in order to understand how IBSA impacts women’s everyday lives and investigates the barriers preventing women from achieving justice in the UK criminal justice system. The author conceptualises IBSA as a gendered phenomenon and therefore situates it upon Kelly’s continuum of violence. Findings from the research suggest that there are significant issues with regard to police and legislative responses to IBSA and, as such, the author calls for an overhaul of the criminal justice response, so as to support women in their pursuit of justice.
Jeacock et al. (2024)	UK	N = 5 victim–survivors (1 male and 4 female). Age 28/29 (4 participants) and age 44 (1 participant).	Semi-structured interviews—Qualitative	This article explores victim–survivors’ experiences of cyberflashing. The authors identify cyberflashing as a gendered phenomenon. The results of the study reveal that victim–survivors view cyberflashing as immoral and dehumanising, but they find reasons to excuse it. Participants explained how victim–survivors are often blamed for their experience and, in turn, experience self-blame. However, throughout the interviews, feelings of self-blame reduced due to a variety of reasons, including maturation and relationship experience.
Krieger (2020)	Canada	N = 174 university students (100 men, 74 women). Mean age: 20.5 years.	Online survey using a vignette approach—Quantitative	This thesis accounts for victim–survivors’ experiences of image-based sexual violence (IBSV) and bystander responses. The studies conducted highlight similarities in women’s experiences between physical sexual violence and IBSV. The findings also suggest that current interventions to prevent sexual violence (through the use of bystanders) may also be adapted for IBSV.
Kromann and Flynn (2025)	Australia/international	N = 17 studies.	Scoping review	This scoping review focuses on exploring existing research in relation to bystanders and IBSA. Their main findings relate to difficulties around terminology around IBSA and bystander intervention being gendered, in that there was less intervention when victim–survivors were women and seen as having high sexual agency. Furthermore, most bystanders recognised IBSA as wrong but often did not intervene, and factors such as having empathy for the victim–survivor increased the likelihood of intervention.
Kugler and Pace (2021)	USA	Study 1. N = 1141 adults (547 male, 594 female). Mean age: 47.81 years Study 2. N = 395 adults (194 male, 201 female). Mean age: 49.18 years Study 3. N = 417 adults (185 male, 230 female). Mean age: 44.81 years	Online survey using fictional scenarios of deepfake distributions— Quantitative	This article draws upon three empirical studies assessing public attitudes towards deepfake technology. The main study shows that participants viewed non-consensually created pornographic deepfakes as extremely harmful, but nonpornographic deepfakes were perceived as substantially less harmful. Two follow-up studies show that participants wanted stronger civil and criminal responses to deepfake creation and perceived the distribution of deepfakes to be just as harmful as ‘revenge porn’.
*** Mainwaring (2023)	UK	N = 85 studies (systematic review). N = 35 university students (4 male, 31 female, 18–53 years) (focus groups). N = 376 adults (174 male, 196 female, 4 self-describe, 2 not disclosed). General population ages 18–39 years (experiments/surveys).	Systematic review, focus group studies and three experiments— Mixed methods	This thesis explores how individual, situational and contextual factors impact bystander interventions in IBSA contexts. A mixed methods approach was employed, and findings suggest that predictors of intervention included: responsibility, confidence, being friends with the victim–survivor, victim empathy, and positive social norms towards intervention. Barriers to intervention included fears for safety as well as fears of audience perceptions.
Mckinlay and Lavis (2020)	Australia	N = 122 adults (29 male, 93 female). General population. Mean age: 27.6 years.	Online survey using scenarios of revenge porn—Quantitative	This article investigates perceptions of ‘revenge porn’ victim–survivors. Participants were presented with ‘revenge porn’ scenarios with varying levels of nudity. The results suggest that victim–survivors who were more naked were more likely to be labelled as promiscuous and be blamed by participants. Additionally, participants who upheld traditional gender roles were more likely to attribute blame to victim–survivors.
Mclocklin et al. (2024)	UK	N = 31 (male 8, female 21) adult victim–survivors, age 19–52 years.	Interviews—Qualitative	This article incorporates the narratives of 31 UK adult victim–survivors of non-consensual dissemination of intimate images (NCII). Specifically, a focus was placed upon victim–survivors’ decision to disclose and experiences of accessing support. Victim–survivors within the study preferred informal support options. The authors identified several barriers to help-seeking, including the stigma attached to NCII and the inaccessibility of formal support services.
Morris (2017)	USA	N = 514 undergraduate students (147 male, 367 female). Age 18–49 years.	Online questionnaire using vignettes—Quantitative	This thesis addresses the extent to which victim gender, a history of infidelity, empathy instructions and observer gender work impact the levels of blame attributed to victim–survivors and perpetrators of revenge porn. The results show that men were more likely to blame the victim–survivor and show empathy towards the perpetrator. Participants in general were more likely to empathise with the perpetrator if the victim–survivor had a history of infidelity.
Ragen (2023)	USA	N = 321 adults (174 male, 147 female). General population, mean age: 35.09 years.	Online survey using scenarios of revenge porn—Quantitative	This study explores the relationship between victim blaming of revenge porn victim–survivors and religious fundamentalism. The study predicted that individuals who scored higher in religious fundamentalism would be more likely to blame a victim–survivor of revenge porn, regardless of the relationship length presented in the scenarios.
Rhyner (2017)	USA	N = 609 adults (male 295, female 303). General population, mean age: 32.41 years.	Online questionnaire—Quantitative	This thesis addresses the effects of victim–survivor gender and remorse in relation to victim blaming in the context of non-consensual photo sharing. The findings suggest that when victim–survivors took the photograph themselves, they were blamed more than those whose photograph was taken by the perpetrator. Men were more likely to blame the victim–survivor than women.
** C. R. Serpe (2020)	USA	N = 359 adults (188 female, 171 male). General population, age 18–71 years.	Online survey using vignettes—Quantitative	This thesis explores the relationship between objectification and victim blame in the context of revenge porn against sexually diverse populations (bisexual, lesbian, straight). The participants themselves were straight, cisgender men and women over the age of 18. The findings suggest that objectification and victim blame are significantly related, but there were no significant differences in regard to the sexuality of the victim–survivor. Men were more likely to blame the victim–survivor in the vignettes provided than women.
Starr and Lavis (2018)	Australia	N = 168 adults (male 29, female 139). General population ages 18–67 years.	Online survey using telephone conversation scenarios relating to revenge porn—Quantitative	This study incorporates revenge porn scenarios in order to explore victim blaming, particularly when revenge porn is perceived as a betrayal. The study used three factors to predict perceptions of blame and betrayal: victim–perpetrator relationship length, the medium used for sexting, and the participants’ level of trust in others. The findings show that the medium through which the sexual image was sent did not impact victim blaming/betrayal perceptions. In contrast, the length of the relationship between the victim–perpetrator did impact victim blaming, but not perceived betrayal.
Uhl (2017)	USA	N = 692 adults (262 male, 406 female, 1 transgender male and 23 no response). General population, ages 18–75 years.	Online questionnaires using vignettes, including a mock police report—Quantitative	Utilising a mock police report referring to a case of non-consensual pornography, this thesis seeks to understand the impact of a victim–survivor’s gender, weight and photograph origination on participants’ attributions of blame. Participants were more likely to blame female victim–survivors when a former partner had taken the photograph. Participants believed that cases were more severe where female victim–survivors had taken the photograph themselves, in comparison to male victim–survivors.
Woolford (2019)	USA	N = 43 college students (8 male, 17 female and 18 not recorded). No ages included, but does include an exclusion question to ask if participants are 18 years or older.	Online survey—Quantitative	This thesis explores victim blaming in relation to sextortion. Specifically, it examines the extent to which participants’ gender and political ideology influence their victim-blaming attitudes. The results show that men were more likely than women to blame victim–survivors of sextortion, and those with conservative political views were also more likely to attribute blame to the victim–survivor.
Zauner (2021)	UK	Case study, N = 3 UK educational campaigns (Exposed, Sexting, Just Send It).	Case study of educational campaigns using media discourse analysis—Qualitative	This article presents a feminist case study of three UK educational campaigns (Exposed, Sexting, Just Send It). In doing so, the authors demonstrate that these campaigns reproduce symbolic violence through victim blaming. For instance, they suggest that victim–survivors are consistently held responsible for their own safety and blamed for their victimisation. Additionally, they suggest the severity of IBSA has been neglected within these campaigns. As such, they call for empirically informed education to help challenge the stigma attached to IBSA.

* Included in review but duplicate data; Flynn et al. (2022b). ** Included in review but duplicate data; C. Serpe and Brown (2022). *** Included in review but duplicate data; Mainwaring et al. (2024, 2025).
